# Non-Additive Coupling Enables Propagation of Synchronous Spiking Activity in Purely Random Networks

**DOI:** 10.1371/journal.pcbi.1002384

**Published:** 2012-04-19

**Authors:** Raoul-Martin Memmesheimer, Marc Timme

**Affiliations:** 1 Department for Neuroinformatics, Radboud University Nijmegen, Nijmegen, Netherlands; 2 Network Dynamics Group, Max Planck Institute for Dynamics & Self-Organization, and Bernstein Center for Computational Neuroscience, Göttingen, Germany; Université Paris Descartes, Centre National de la Recherche Scientifique, France

## Abstract

Despite the current debate about the computational role of experimentally observed precise spike patterns it is still theoretically unclear under which conditions and how they may emerge in neural circuits. Here, we study spiking neural networks with non-additive dendritic interactions that were recently uncovered in single-neuron experiments. We show that supra-additive dendritic interactions enable the persistent propagation of synchronous activity already in purely random networks without superimposed structures and explain the mechanism underlying it. This study adds a novel perspective on the dynamics of networks with nonlinear interactions in general and presents a new viable mechanism for the occurrence of patterns of precisely timed spikes in recurrent networks.

## Author Summary

Most nerve cells in neural circuits communicate by sending and receiving short stereotyped electrical pulses called action potentials or spikes. Recent neurophysiological experiments found that under certain conditions the neuronal dendrites (branched projections of the neuron that transmit inputs from other neurons to the cell body (soma)) process input spikes in a nonlinear way: If the inputs arrive within a time window of a few milliseconds, the dendrite can actively generate a dendritic spike that propagates to the neuronal soma and leads to a nonlinearly amplified response. This response is temporally highly precise. Here we consider an analytically tractable model of spiking neural circuits and study the impact of such dendritic nonlinearities on network activity. We find that synchronous spiking activity may robustly propagate through the network, even if it exhibits purely random connectivity without additionally superimposed structures. Such propagation may contribute to the generation of spike patterns that are currently discussed to encode information about internal states and external stimuli in neural circuits.

## Introduction

Patterns of spikes that are precisely timed within the millisecond range have been investigated and observed in a series of neurophysiological studies [1]–[9]. This supports the ongoing debate whether cortical neurons are capable of precisely coordinating the timing of their action potentials across recurrent networks and whether only the neurons' firing rate or also the precise timing of their spikes encode key information that is intimately related to external stimuli and internal events [2], [3], [10]–[14].

During the last two decades, a branch of theoretical research has focused on the question how such precise timing could emerge. One prominent, possible explanation for the occurrence of precisely coordinated spiking is the existence of excitatorily coupled feed-forward structures, ‘synfire-chains’, which are superimposed on a network of otherwise random connectivity, e.g. through strongly enhanced synaptic connectivity [10], [15]–[18]. Under certain conditions, these additional feed-forward structures enable the persistent propagation of groups of spiking activity that is synchronous on a time scale of down to one millisecond [17], [19]–[24]. So far, however, experimental research did not provide anatomical evidence for such structures. Other studies proposed that asynchronous propagation along paths with matching inhomogeneous delays [25] or the dynamics of local recurrent networks [26], [27] might underlie precisely timed spike patterns.

Here we show that nonlinear dendritic interactions, recently uncovered in neurophysiological experiments, offer a viable mechanism to support stable propagation of synchrony through random cortical circuits without additionally superimposed structures: Excitatory synaptic stimuli may not only superimpose linearly or sublinearly [28], [29], but may also induce strongly nonlinear, supra-additive coupling enhancement due to dendritic spikes [30]–[32]. Fast dendritic sodium spikes strongly enhance the effects of stimulus-evoked post-synaptic potentials in a supra-additive way and induce precisely timed and sharply peaked depolarizations in the somatic membrane potential. Remarkably, this enhancement occurs reliably only if the stimuli are synchronous in time with temporal difference of less than 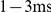 [33]–[36], cf. also [37]. If the resulting depolarization triggers an action potential, it is highly precise in time up to less than 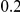 ms [33]–[35]. Other types of much slower dendritic spikes are mediated by voltage gated 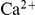 or NMDA channels. They have longer time courses up to several hundreds of milliseconds and do not depend on synchronous stimulation (see, e.g., [38], [39], and, for reviews, [32], [40]).

In the following, we study consequences of coupling nonlinearities that are due to fast dendritic spikes onto the collective dynamics of recurrent neural networks. We find that, in contrast to linearly coupled networks, propagating synchronous activity may persist already in networks of simple neurons that have purely random connectivity and exhibit no additional structures. We conclude that the characteristic features of dendritic nonlinearity, in particular the amplification of (only) synchronous input and the induction of temporally precise output, predestine them to support the generation and propagation of persistent, highly synchronous spiking activity.

## Results

### Neurons coupled via nonlinear dendrites

We investigate networks of integrate-and-fire neurons in the limit of fast response to incoming spikes and with nonlinear interactions (see Methods). Similar models with linear interactions are widely used for studying the dynamics of networks of spiking neurons (see, e.g., [16], [41]–[43], [44], [45] for recent reviews) because they capture essential features of cortical neurons and at the same time allow to investigate the mechanisms underlying the dynamics of networks without obscuring them by a many-parameter, many-variable single neuron description (see, e.g., [44], [46]–[48]). In this study they allow to interpret the dynamical regimes of the network activity qualitatively and to analytically assess them quantitatively. We assume that the delay 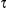 between sending of a spike by a presynaptic neuron and postsynaptic (somatic) response is identical for all neurons. This is appropriate for the description of responses mediated by fast dendritic spikes because these evoke a fast and precise rise with sub-millisecond rise time constant in the somatic potential [33], [36]. Moreover, if a somatic action potential is generated by fast dendritic spikes as observed in [33], this occurs 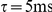 after presynaptic axonal stimulation with only sub-millisecond inter- and intra-neuronal jitter, while the action potential timing strongly varies in time if no dendritic spike is elicited. This is well resembled by our model dynamics where nonlinearly enhanced inputs yield fast, jump-like responses in the membrane potential and firing due to supra-threshold excitation occurs precisely after the delay time 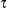. For simplicity, we further assume that all postsynaptic responses to spikes occur after this delay time. ‘Imprecise’ spiking is generated due to a constant supra-threshold input current.

**Figure 1:**
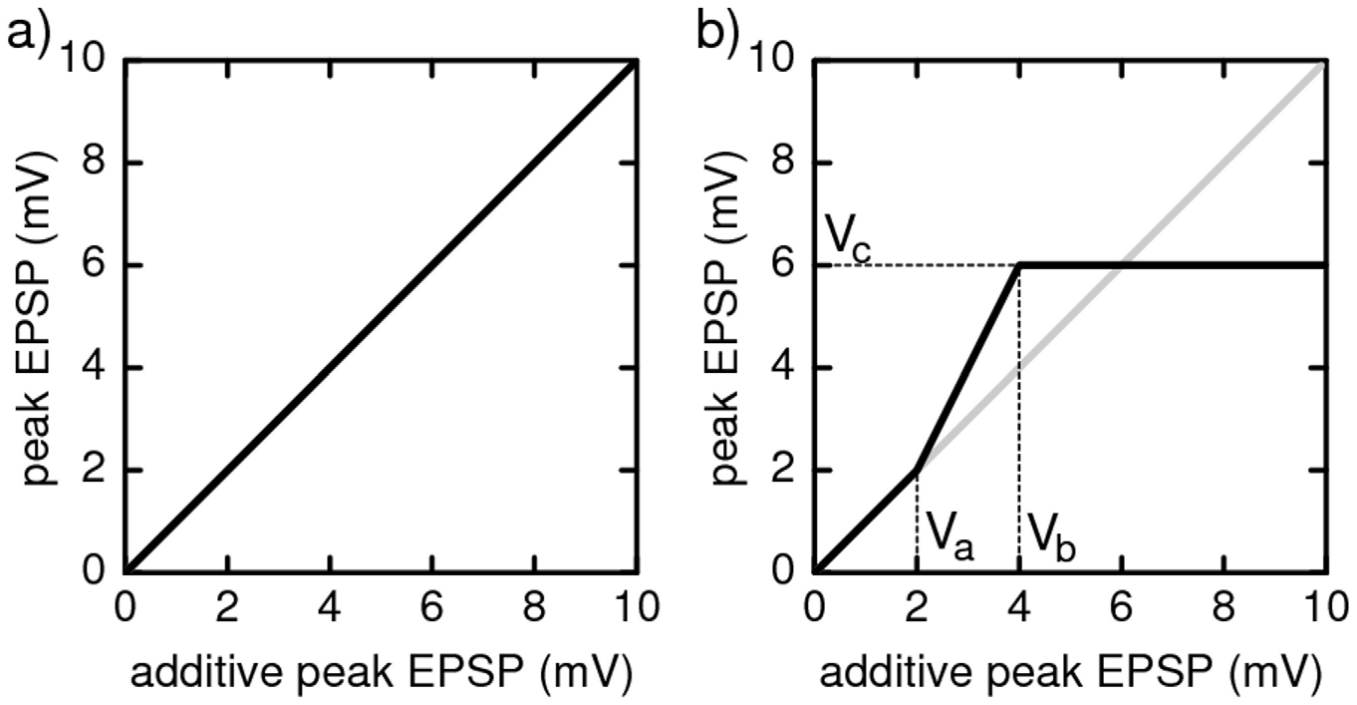
Dendritic modulation function for (a) additive and (b) non-additive coupling. The modulation function maps the somatic peak EPSP expected from linear summation of inputs to the actual peak EPSP strength. In networks with additively coupled neurons (a), the modulation function is the identity. In networks with nonlinear dendritic enhancement of inputs (b), the modulation function is sigmoidal as found in physiological experiments. Supra-additivity sets in when the expected (linearly added) input strength reaches a threshold 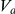; at some strength 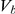 the response saturates at a level 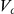.

To account for nonlinear enhancement and saturation of synchronous excitatory inputs, we modulate the linear sum of the amplitudes of excitatory post-synaptic potentials (EPSPs) that arise simultaneously from different synapses by a nonlinear function 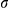. This covers the main features of experimentally found nonlinear dendritic amplification (cf. [33], [36], [38]–[40]), thus effectively modeling a neuron with one, nonlinear dendrite. For the neuron model considered, 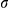 has a straightforward interpretation: It maps the peak EPSP amplitude 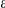 expected from linearly adding the coupling strengths of synchronously received excitatory signals to the actual value 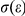 (cf. Fig. 1b). Such a modulation function has been directly [39] and indirectly [33] measured in experiments. It has a sigmoid shape, with linear summation for small summed amplitudes 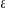 and saturation at high 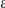. We thus model the non-additive coupling using a function 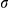 that is the identity 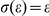 at low values 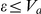, has a constant saturation 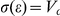 at high values 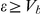, and linearly interpolates in between, cf. Fig. 1b. Inhibitory post-synaptic potentials (IPSPs) at the same neuron are linearly summed, independent on whether or not the synaptic signals are simultaneous, because there is no experimental evidence for supra-linear enhancement. If 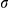 is the identity function (Fig. 1a), the same holds for excitatory coupling and we recover a “conventional” network of linearly coupled neurons.

### Propagation of synchrony

In both additively and non-additively coupled sparse random recurrent networks, asynchronous irregular spiking activity constitutes a dynamical state typical for a wide range of parameters [42], [43], [49], [50]. Sequences of groups of synchronously spiking neurons may spontaneously occur starting with a single neuron, or they can be initiated by a group of neurons that was excited to synchronous spiking by external input. If a single neuron or a group of neurons send spikes at one given time, a subset of neurons in the network will receive a synchronous pulse of spikes a delay time 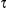 thereafter. All neurons for which the induced postsynaptic response leads to a supra-threshold depolarization in turn spike simultaneously so that another synchronous pulse of spikes is generated which can excite a further group of neurons and so on. Spontaneous chains are part of the background activity. They usually involve only small numbers of synchronously spiking neurons and quickly extinguish, cf. supporting Fig. S1.

**Figure 2:**
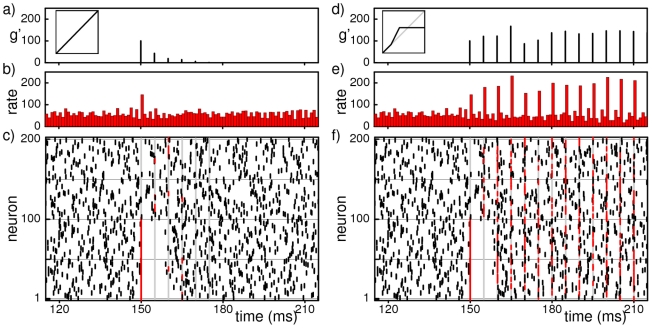
Non-additive coupling enables persistent propagation of synchronous spiking. The figure illustrates the temporal evolution of propagating synchrony as typical for large ranges of parameters in conventional networks (a,b,c) and in networks incorporating nonlinear dendritic interactions (d,e,f). Panels (c,f) show the spiking activity of the first 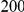 neurons in a network of 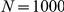 neurons versus time. A chain of synchronous pulses is initiated by applying external supra-threshold inputs to the first 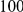 neurons at time 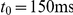 (red colored spikes, grey vertical lines indicate times where spikes occur as part of the chain). Panels (a,d) show the total size 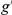 of synchronized groups within the chain. In the linearly coupled network, the chain of synchronous activity extinguishes after a few steps. In the network with nonlinear dendritic integration, synchronized spiking activity propagates persistently. The presence of large synchronous pulses is reflected in the network rate, see panels (b,e) (rate in kHz, bin size 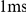).

**Figure 3:**
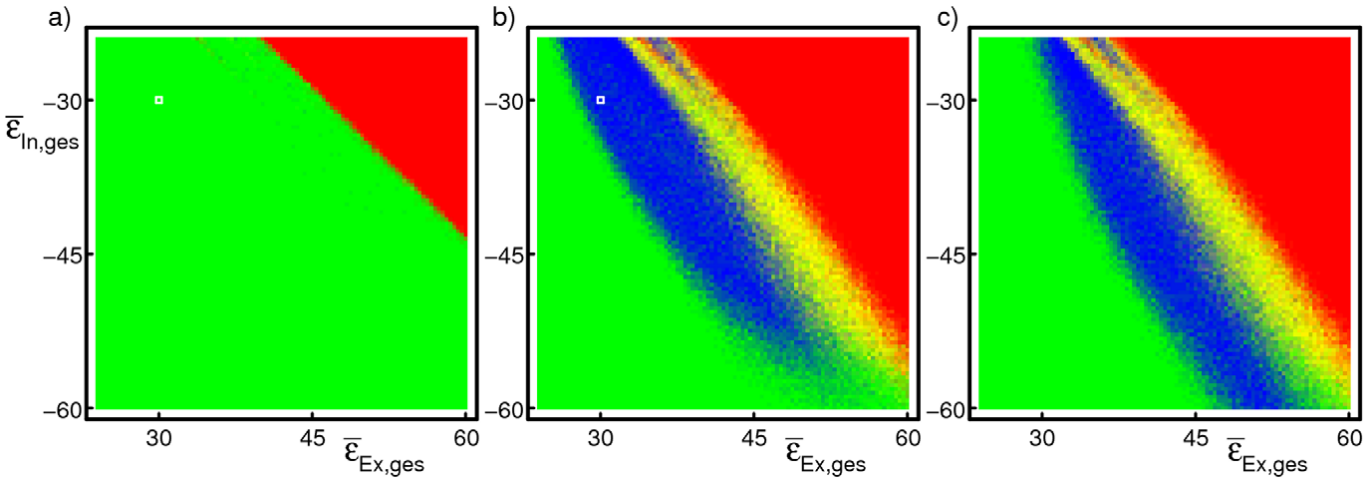
While propagation of synchrony is usually short-lived in linearly coupled networks (a), it is persistent for a wide range of coupling parameters if the neurons are nonlinearly coupled (b,c). The parameter scans illustrate this by varying the mean total input strengths 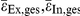 of the excitatory and the inhibitory input in a network of 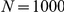 neurons with 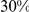 connectivity. For each combination, synchronous activity was initiated with 100 neurons (a,b) or 75 neurons (c) and the stability of the temporal evolution was assessed. Blue coloring indicates stable propagation of synchrony, red and yellow coloring refers to unstable background activity before and after onset of propagation, and green coloring indicates unstable propagation (see Methods for details). White squres in (a) and (b) indicate the coupling strengths employed in Fig. 2a) and b), respectively. The large blue areas in the scans for nonlinearly coupled networks indicate that propagation of synchrony is stable in a wide range of parameters for such networks. This area is absent for linearly coupled networks as shown in (a), for smaller initial pulse sizes (e.g. 75 neurons) the number of successful trials is even smaller. In nonlinearly coupled networks with larger coupling strengths, an initial pulse size of 100 neurons can be larger than the upper bound of the propagation zone so that the chain is unstable (b) while for the same coupling parameters an initial pulse of size 75 neurons starts a stable chain (c). For smaller coupling strengths, an initial pulse size of 75 neurons can be insufficient to initiate stable propagation in contrast to a pulse of size 100 neurons.

How does a sparse random network respond to induced synchronous activity, initiated, e.g., by external stimuli? We compared the responses in networks with purely linear, additive coupling to those where the excitatory inputs cooperate supra-additively. For linearly coupled networks we find that pulse sizes in chains of synchronous spiking activity quickly reduce to the level of spontaneous synchronization and the chains rapidly die out (cf. Fig. 2a). Propagation of synchrony is therefore short-lived in linearly coupled networks, consistent with previous studies [16], [17], [51]. In contrast, for nonlinearly coupled networks, in a wide range of parameters (cf. Fig. 3), a chain initiated by a large enough, but not too large synchronous group after a few steps reaches pulse-sizes that fluctuate around some typical value, Fig. 2b. These sizes are substantially larger than the sizes of synchronous pulses occurring in the background activity (cf. Fig. S1b), which persists while synchrony is propagating on top of it. Only if the initial group size is too large, the chain of synchronous activity is again short-lived. Taken together, we find *persistent* propagation of synchrony in non-linearly coupled networks.

Persistent propagation of synchrony is robust against parameter changes. We estimate a range of coupling strengths where persistent propagation of synchrony occurs in linearly and in nonlinearly coupled networks in Fig. 3. Background activity is here considered stable if it contains at no time any synchronous pulse of more than 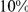 of the network size (red coloring if it became unstable spontaneously, i.e. before initiation of synchronous activity, yellow coloring if it became unstable thereafter, cf. also supporting Fig. S1). Propagation of synchrony is considered persistent if background activity is stable and if at least 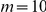 synchronized groups within the chain are distinguishable from background activity, i.e. the minimal group size 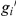, 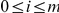, is larger than the largest group size occurring in background activity (green coloring for stable background activity but short-lived propagation of synchrony, blue coloring for stable background activity and persistent propagation of synchrony). In nonlinearly coupled networks, propagation of synchrony is persistent in a wide range of parameters, while it is usually short-lived in linearly coupled networks.

The mechanisms underlying this persistent propagation of synchrony can be intuitively understood. Sequences with small groups of synchronized neurons behave as for linear, additive coupling, i.e. they usually extinguish after a few steps, so there is no persistent spontaneous propagation and irregular background dynamics for the entire network is stable. If larger groups of neurons send spikes simultaneously, their postsynaptic neurons receive sufficiently many excitatory inputs so that the nonlinearities become effective. Since the inhibitory couplings add only linearly, excitatory input surpasses inhibitory input for a larger fraction of postsynaptic neurons than in a linearly coupled network. This causes more neurons to fire in response to the synchronous pulse; the number of neurons synchronized in each step of the chain grows. If synchronous pulses become too large, saturation becomes important and excitation becomes less efficient compared to inhibition. Further, many neurons are refractory. This implies that less neurons are excited in response to overly large groups of synchronously spiking neurons; consequently the group size is reduced. In addition, fluctuations in groups sizes occur due to the randomness of the network connectivity and the distribution of membrane potentials during pulse reception. These qualitative mechanisms keep the group sizes substantially large and fluctuating within a certain range.

### Quantitative analysis of the non-propagating and the propagating state

**Figure 4:**
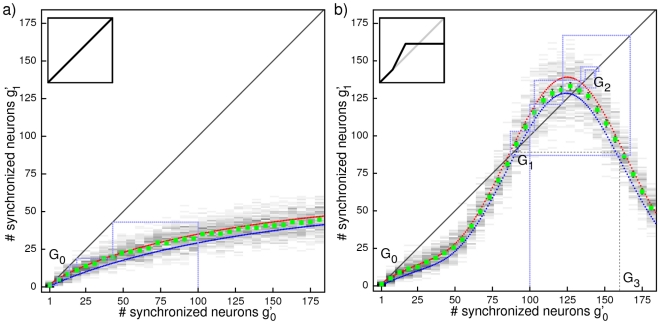
Evolution of synchronous pulses in linearly (a) and nonlinearly (b) coupled networks. Numerically derived probability distributions 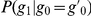, i.e. probabilities of pulse-sizes 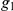 in response to a pulse of size 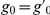 are shown by gray shading; associated conditional expectations 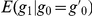, i.e. numerically derived mean response group sizes, are displayed by green squares. Error of the mean (confidence intervals: two times standard deviation) has about the size of the plot symbol, larger errors are given by error bars. Analytical results for 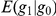 derived from diffusion approximation and statistics of the underlying network topology are given by blue dots, results from a semi-analytical approach are given by red dots. Assuming stationarity and the Markov property, the probability distributions can be interpreted as stochastic iterated map or transition matrix for the pulse-sizes in a chain of propagating synchronous activity. For linearly coupled neurons, there is no area from where the pulse-sizes do not quickly converge with high probability to the level of spontaneously synchronized neuron groups. As an explicit example, the light blue dotted lines display the dynamics from Fig. 2a as result of graphical iteration using the stochastic iterated map. In contrast, for nonlinearly coupled networks, the probability for chains with pulse-sizes between 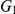 and 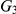 to converge to the level of spontaneously synchronized neuron groups is rather low: There is a state of persistent propagation in the network located around 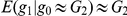. As an explicit example for dynamics assuming this state, the light blue dotted lines display the chain from Fig. 2b as a result of graphical iteration.

To quantitatively understand the mechanisms underlying persistent propagation of synchrony and to determine the group sizes which initiate and take part in persistent propagation, we studied the evolution of propagating synchrony both analytically and numerically (see Methods and Fig. 4). Approximating the dynamics of group sizes by a Markov process, we derived the transition probabilities 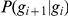 for the transitions from the sizes of the 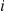
th pulse to those of the 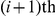. Here 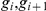, 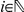, are random variables that assume values in 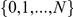, where 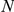 is the number of neurons in the network. Accordingly, 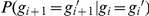 is the probability that the 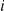
th pulse generated by 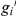 simultaneously spiking neurons causes a group of 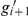 neurons to spike simultaneously in response. From the conditional (transition) probabilities, we derived the conditional expectation 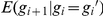, i.e. the average size of a pulse following a pulse of size 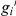. Since the distributions 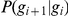 are similar to 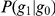 also for later stages 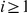, we assume stationarity and approximate 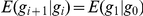 and 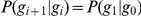 for all stages 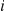 of propagation. The points 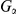, 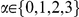, where 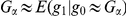 for 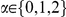 and 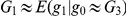, 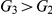, determine the range of typical group sizes occurring in the networks (Fig. 4). The analytical predictions agree well with the numerical results. The quantities 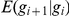 and 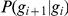 yield a quantitative explanation of the mechanisms that lead to persistent propagation of synchrony:

For networks of linearly coupled neurons, each synchronous group with 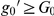 neurons (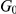 small, e.g. 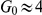 in Fig. 4a) on average excites synchronous groups with less neurons. The smaller groups in turn excite even smaller groups so that synchronous activity rapidly decays to the level of a few synchronized neurons and fluctuates near 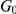. Thereafter, due to the fluctuations from the already small group size, propagating synchronous activity rapidly extinguishes completely (group size zero). So the theory predicts that in networks of linearly coupled neurons the chain of synchronous activity quickly extinguishes even if excited by external synchronous input, consistent with the above observations (Fig. 2a). Since the shape of the transition matrix stays invariant when network parameters like the coupling strengths are changed, such a change will not lead to persistent propagation of synchrony. If, e.g., the size of excitatory coupling strength is increased, only the slope of the curve is increased. This predicts the transition to unstable background activity shown in Fig. 3.

In contrast, nonlinear supra-additive excitatory coupling enables persistent propagation of activity with a substantial number of neurons synchronized. The sizes of the propagating synchronous pulses are of the order of a typical size 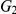 and range between 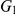 and 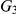, all of which are substantially larger than 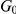 (cf. Fig. 4b). Pulses of sizes between 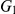 and 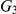 usually evoke pulses of sizes in the same range, i.e. between 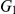 and 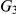 again. Only rarely, propagating synchronized activity becomes smaller than 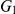 or larger than 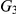; if so, the pulse size is likely to stay smaller than 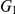 for longer, decay even further as for linearly coupled networks, and the chain may cease to exist. A steeper and narrower peak can lead to transiently increased activity and short-lived propagation of larger synchronous groups [51].

The different dynamics for linearly and nonlinearly coupled networks can also be understood by approximating the stochastic dynamics by a deterministic iterative map derived from interpolating between the values of 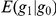. For networks of linearly coupled neurons, the map has only one stable fixed point 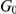 which is at small pulse sizes of the order of spontaneous synchronization; it may be distinct from the trivial fixed point zero. Any larger initial pulse size will thus lead to a chain decaying to the level of spontaneous synchronization. If coupling is non-additive, there can be two stable fixed points 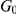 and 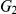, and an unstable fixed point 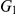 in between. Chains starting with sizes in the basin of 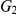 between 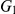 and 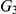 then evolve towards stable propagation with pulse-size 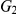. For different parameter settings, stable propagation of synchrony is supported by a stable periodic orbit close to an unstable fixed point 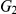.

Taken together, the theory for nonlinearly coupled networks predicts persistent propagation of synchronous activity in a typical range of pulse sizes and a decay that is possible only due to fluctuations. This agrees with the numerical observations (Fig. 2b).

## Discussion

In summary, we presented a theoretical analysis and numerical simulations of recurrent networks of spiking neurons with nonlinear dendritic interactions. The results indicate that networks with nonlinear dendritic interactions are capable of generating persistent propagation of synchronous spiking activity even if the network is purely randomly connected and has no additional structural features.

Theoretical studies on active dendrites mainly considered single neurons. Simulations of neuron models with detailed channel density and morphology showed dendritic spike generation in agreement with neurobiological experiments [33], [34], [36], [38]. For neurons with slow dendritic spikes, which are largely insensitive to temporal coincidence of inputs, firing rate models have been developed [52]. They reproduce the response properties of detailed models to diverse stimuli and possess computational capabilities comparable to multi-layered feed-forward networks of simple rate neurons [38], [39]. Based on this result, the computational abilities of simple circuits have been considered, also with other types of neuron models (e.g. [32], [53], [54]). Refs. [55], [56] studied propagation of bursts in networks where the bursts can be explained by slow dendritic spikes, and slow nonlinear dendrites were suggested to underlie the persistent activity observed in working memory tasks [57]. Active dendrites generating fast dendritic sodium spikes were studied in a two-neuron circuit and in a simple feed-forward structure [58], and model neurons incorporating such dendritic spikes were used as an output layer in simulations of hippocampal network models [59]. Very recently, ref. [51] has shown that fast dendritic spikes can lead to intermittent, transiently increased propagation of synchrony and it was suggested that they underlie hippocampal sharp wave/ripples characteristic for slow wave sleep.

The present study now shows that fast dendritic spikes can lead to *persistent* propagation of synchrony in random neural networks. In particular, feed-forward structures based on large-scale additional couplings [10], [15], [16] or strongly and systematically adapted strengths of specific synapses and neuron properties [17] may not be needed. As such, our results suggest an alternative mechanism and a potential complementary explanation for the occurrence of patterns of precisely timed spikes [1]–[5], [7]–[9].

Our study uses a model that is appropriate for quantitative numerical analysis of larger networks and at the same time allows analytical predictions that yield further insights into the dynamics of recurrent networks. The theoretical predictions made are based on mean field arguments, strictly valid only in the limit of infinite network size [42], [49], [60]. As our results indicate, these predictions are in good agreement with simulation data already for networks of finite size. The number of neurons participating in pulses of synchronous activity as well as their number relative to the total number of neurons may vary strongly with network features such as the connectivity and the effective total input coupling strengths. Additional external noise, e.g. due to further random spiking inputs, is expected to be beneficial because it stabilizes background activity and leads to a fast equilibration of the neurons' potentials after a synchronous pulse. Both facts support dynamical mixing and thus are in favor of our approximation that the propagation of synchronous activity does not further influence the statistics of the background. We have demonstrated that nonlinear dendritic interactions enable persistent propagation of synchrony even in random neural networks. The results show that the nonlinear interactions are in fact the main ingredient controlling the mechanism underlying the transition to persistent propagation (Fig. 4a vs. 4b), so that the phenomenon is insensitive against variations in parameters such as details of the individual neuron dynamics, the exact form of nonlinearly modulated interactions (Fig. 1), and the coupling strengths (see Fig. 3).

The current study contributes to a new field of research that focuses on neural networks with supra-additive coupling. The influence of different levels of individual neuron reliability, of recurrent and feed-forward network topologies, of dynamic connectivity (learning) and of slow dendritic spikes have to be reconsidered in this context. Our study also suggests future experiments on the propagation of synchrony due to nonlinear dendritic interactions e.g. in cultured neurons [61]. Interestingly, the propagation of synchrony found here for nonlinearly interacting neurons does not follow any specific, predefined propagation paths of synchronous activity across the network; the propagation path will depend not only on the currently excited group but also on which neurons in the background activity are sufficiently depolarized when they receive synchronous spikes from the current group. In a random network, the propagation of synchrony will thus resemble reverberating high-frequency oscillations involving highly synchronous spiking activity. The network structure might shape the activity and lead to a significantly enhanced occurrence of specific sequences of synchronous groups. These spike patterns, however, are noisy and less obvious than those in synfire-chains [10], [15]–[17], [19]–[21], [23], [24], where the propagation paths of synchronous activity are predefined by the embedded feed-forward networks. These different dynamics may provide an experimentally testable distinction between synchronous events created by synfire chains via additional feed-forward structures and those created by nonlinear dendritic interactions in largely or purely random networks. Of course, a more specifically structured network connectivity [62]–[64], the effects of synaptic location on different dendritic branches [39], specific distributions of transmission delays [25], [65]–[67] as well as strongly heterogeneous synaptic strengths [17] will further influence pulse propagation. As an example, nonlinear interactions may facilitate or enable localized persistent synchrony in Hebbian cell assemblies [18], [68], [69]. It will thus be important to extensively investigate to which degree nonlinear interactions as well as non-random network structure are contributing to creating collectively coordinated spiking dynamics, in order to understand the computational capabilities of cortical networks.

## Methods

### Neural network model

We considered networks of 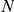 leaky integrate-and-fire neurons connected to form an Erdös-Rényi random graph [70] where each directed synaptic connection between two neurons is present independently with probability 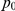. For each connection, the probabilities 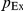 and 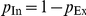 specify whether the coupling is excitatory or inhibitory. The dynamics of the membrane potential 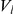 of neuron 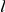 obeys
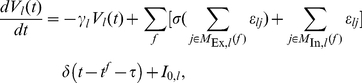
(1)where 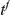 denotes times at which spike are sent within the network, the inverse membrane time constant 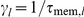 measures the dissipation of the neuron and 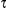 is the transmission delay. We further introduced the set
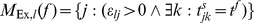
(2)of neurons sending at time 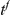 an excitatory spike to neuron 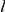, where 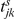 is the 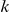
th spike time of neuron 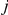 and 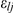 is the coupling strength from neuron 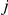 to neuron 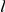. The set
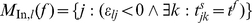
(3)lists the neurons sending at time 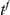 an inhibitory spike to neuron 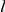. 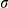 is the possibly nonlinear dendritic modulation function mapping the input strength expected from linear addition of excitatory inputs to the actual input strength. Each neuron receives some constant external input 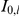. When the membrane potential reaches or exceeds the threshold, 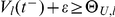, where 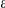 is the possibly arriving total input at time 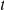, it is reset to 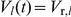 and a spike is emitted. See supporting Table S1 for a tabular description of our model following ref. [71].

The parameters used in the given examples are 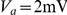 for the onset of supra-additivity, 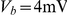 for the onset of saturation and 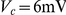 for the level of saturation, in agreement with a direct experimental measurement of 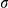 given in [39] for slow nonlinear interactions. In [33], the onset of nonlinearity and the level of saturation lie higher. For comparison with linearly coupled networks, we take an identity 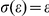 modulating function, effectively choosing 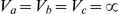, i.e. there is no supra-additivity and no saturation. The analytical methods presented below and the theory presented in the main text are valid for arbitrary parameter choices and hold as long as the background activity stays asynchronous, irregular and sufficiently uncorrelated. In the simulations, the remaining network parameters are 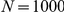, 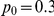, 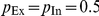, 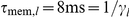, 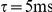, 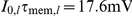, 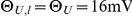, 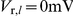. If not stated otherwise, 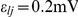, if the coupling strength from neuron 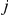 to neuron 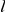 is excitatory and 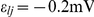, if it is inhibitory.

### Numerical methods

Network simulations were done in phase representation [72]. For this, the membrane potential 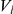 and its threshold 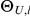 are mapped one-to-one to a phase 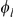 and a phase-threshold 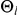 using the inverse of the transfer function 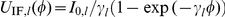 of the leaky integrate-and-fire neuron, as elaborated in ref. [27]. 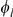 evolves linearly with slope 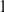 between spike sendings and spike receivings. Spike sendings occur when the phase reaches or exceeds its threshold 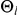. When neuron 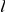 receives input of total strength 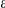 at time 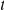, its phase 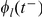 is updated according to 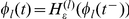, where 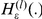 is the response function of the leaky integrate-and-fire neuron, 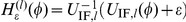 for subthreshold total inputs 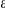 and 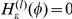 for suprathreshold ones which evoke spike sending.

The numerical simulations were implemented using an event based algorithm which may be outlined as follows [41], [50], [73], [74]: We keep track of the “pseudo-spike time” [75] of each neuron 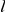, i.e. of the time 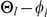 remaining to the next hypothetical spike of the neuron without interaction. Further, we keep track of the spike arrival times together with the neurons that sent the spikes. In each step, the smallest pseudo-spike time is compared with the time remaining until the next spikes arrive. If the next event is (i) a spike sending event, the dynamics is linearly evolved to this event and the pseudo-spike time of each sending neuron 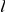 is reset to 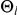. The newly sent spikes are stored in the spike list. If the next event is (ii) a spike receiving event, the dynamics is linearly evolved to this event and the excitatory and inhibitory input strengths to each neuron 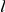 are determined. We apply 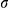 to the excitatory input strength and add the inhibition. The resulting total input strength 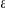 determines the update of the phase via 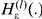 and therewith the new pseudo-spike time as well as immediate spiking responses.

For the spike-train analysis, propagating chains initiated at some time 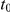 can be separated from background activity because synchronized groups which are part of the chain by construction send spikes precisely at 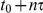, 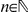, while spikes which are part of background activity are sent at times which are at least slightly different.

Fig. 4 shows the numerically derived frequency of occurrence of a group size 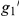 when the initial group had size 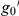 and its mean value, which are approximations to the conditional probability 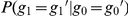 and the conditional expectations 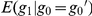, respectively. For the numerical measurements, synchronous pulses of size 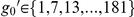 were initiated twice after equilibration of the dynamics (initial phases were randomly drawn from a uniform distribution on 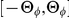 where 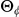 is the phase threshold, and 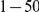 random initial spikes were added) in 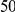 different random networks and the size 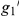 of the subsequent pulse was measured. Fig. 2 shows two single simulations with 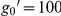.

For Fig. 3, the mean total input strengths 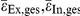 of the excitatory and the inhibitory input were varied in steps of 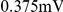 by changing 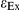 and 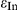, from 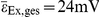 (corresponding to 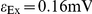) to 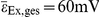 (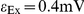) and from 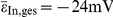 (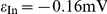) to 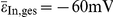 (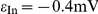). For each data point, the stability of background activity and the persistence of propagating synchrony was checked in 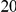 different random networks with different random initial conditions, initial phases were drawn from a uniform distribution on 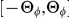 where 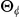 is the phase threshold, and 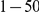 random spikes initially in transit were added. The stability of background activity without propagating synchrony was checked for simulated time 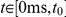, where 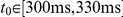. At 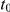, synchronous activity was initiated by external stimulation of a group of 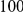 neurons. Stability of propagating synchrony was checked for 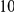 steps after initiation (corresponding to 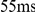 of propagation) and stability of background activity after 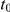 was checked for an interval of 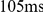 after pulse initiation. We note that for stable irregular background activity finally (for time tending to infinity) every chain will die out with probability one, because the group size has finite probability to leave the zone of propagation and to reach the absorbing fixed point zero.

We implemented the network dynamics simulations in C and embedded them with MathLink into Mathematica. We used Mathematica to implement user interfaces, control programs and data analysis.

### Analytical methods

We computed the transition probabilities for the group-sizes analytically and semi-analytically. In the analytical approach, the probability distribution for the membrane potentials 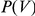 was derived in diffusion approximation, also approximating the actual number of synaptic connections by its mean and describing the background activity as consisting of independent Poissonian spike trains [42], [44]. To eliminate errors due to these approximations in a semi-analytical approach, 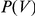 was derived by direct measurements of the relative frequency of occurrences of membrane potentials at different times in 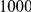 numerical network simulations, 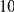 simulations in 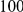 different random networks with different random initial conditions as described above. In both approaches, we computed from 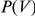 the cumulative probability distribution from the right,
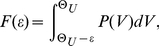
(4)which yields the average probability 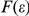 that a neuron is excited above threshold when it receives an input of strength 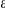. We further assumed (a) that previous groups with 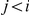 do not influence 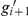, i.e. the sequence of group sizes is a realization of a Markov chain, (b) that the propagating synchrony does not change the statistics of the background dynamics of the non-participating neurons, and (c) that neurons which spiked in the 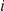
th step are refractory while the other neurons are equilibrated at the time of the 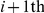 pulse. The validity of the approximations depends on the network parameters and was checked by numerical simulations. Under these assumptions, the statistical properties of the neural network topology allow to compute the probabilities that a neuron receives an input of strength 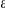 at time 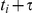 under the condition that a synchronized group of size 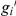 has sent spikes simultaneously at time 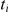. Together with 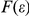, the conditional probability distributions 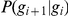 and the conditional expectations 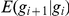 can be derived. 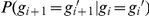, the probability that a group size 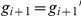 occurs in response to a group size 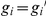, follows a binomial distribution,
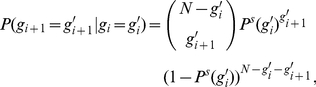
(5)where
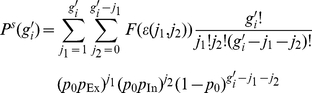
(6)is the probability that a neuron spikes in response to a synchronous pulse of 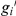 spikes. 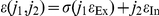 is the total input strength due to 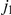 excitatory and 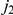 inhibitory inputs and 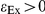 and 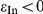 are the strengths of excitatory and inhibitory connections. According to Eq. (5), 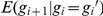, the average next group size 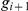 given a current group size of 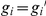, is
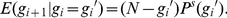
(7)

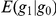
 as derived from the diffusion approximation and from the semi-analytical approach is illustrated in Fig. 4 for linearly and nonlinearly coupled networks. The values agree well with the results of the explicit numerical measurements, deviations are due to the specified approximations. The critical pulse-sizes 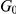, 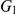 and 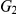 are the intersection points of the interpolated 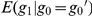-values with the diagonal, 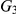 denotes the size 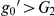, where the interpolated 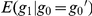-values equal 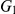. If present, 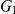 and 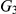 roughly bound the pulse-sizes in persistently propagating chains of synchronous activity.

## Supporting Information

Figure S1Distribution of sizes of synchronous pulses in the background activity, where spikes belonging to the externally initiated propagating chain of pulses have been removed. The distributions are similar in linearly (a) and in nonlinearly (b) coupled networks. The figure exemplarily displays the sizes of spontaneously synchronized pulses in the background activity within the interval 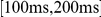 for the dynamics shown in Fig. 2a and 2b in the main text, respectively. While small pulse sizes of the order of 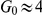 (see Fig. 3 in the main text) are relatively common, large pulses do not occur on relevant time scales. The chain of synchronous activity excited in the linearly coupled network quickly decays to this level of spontaneous synchronization. In contrast, in the nonlinearly coupled network, the pulse-sizes of propagating chains are of the order of 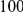 neurons and thus clearly separated from the spontaneously occurring pulses: The propagation of synchrony is persistent.(TIF)

Table S1 Tabular description of our model following ref. [71].
(PDF)
