# Supplementary figures and images for: Non-Additive Coupling Enables Propagation of Synchronous Spiking Activity in Purely Random Networks
Source: PLoS Comput Biol. 2012 Apr 19;8(4):e1002384. doi: 10.1371/journal.pcbi.1002384 (PMC3330086; doi:10.1371/journal.pcbi.1002384)

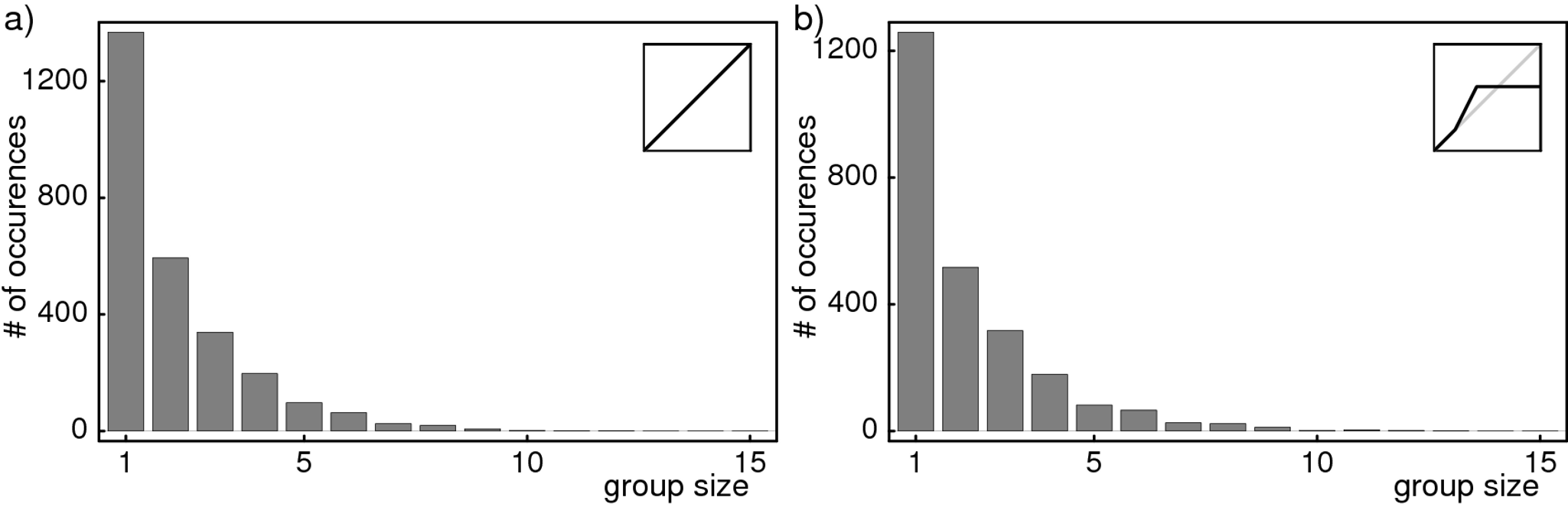

Supplement: Figure S1 — Distribution of sizes of synchronous pulses in the background activity, where spikes belonging to the externally initiated propagating chain of pulses have been removed. The distributions are similar in linearly (a) and in nonlinearly (b) coupled networks. The figure exemplarily displays the sizes of spontaneously synchronized pulses in the background activity within the interval for the dynamics shown in Fig. 2a and 2b in the main text, respectively. While small pulse sizes of the order of (see Fig. 3 in the main text) are relatively common, large pulses do not occur on relevant time scales. The chain of synchronous activity excited in the linearly coupled network quickly decays to this level of spontaneous synchronization. In contrast, in the nonlinearly coupled network, the pulse-sizes of propagating chains are of the order of neurons and thus clearly separated from the spontaneously occurring pulses: The propagation of synchrony is persistent. (TIF) [file pcbi.1002384.s001.tif]
