# Supplementary material for: Non-Additive Coupling Enables Propagation of Synchronous Spiking Activity in Purely Random Networks
Source: PLoS Comput Biol. 2012 Apr 19;8(4):e1002384. doi: 10.1371/journal.pcbi.1002384 (PMC3330086; doi:10.1371/journal.pcbi.1002384)
Supplement: Table S1 — Tabular description of our model following ref. [71]. (PDF) [file pcbi.1002384.s002.pdf]

A	Model summary
Populations	One, excitatory-inhibitory hybrid neurons
Topology	—
Connectivity	Erdös-Rényi random graph
Neuron model	Leaky integrate-and-fire, fixed voltage threshold, no absolute refractory time
Channel models	—
Synapse model	δ-current inputs (discontinuous voltage jumps), additive or supra-additive input summation
Plasticity	—
Input	Constant external input current to each neuron
Measurements	Spiking activity, membrane potentials

B	Population
Elements	LIF neurons
Size	N

C	Connectivity
Type	Sparse, random recurrent, connections randomly chosen to be present with probability p_0
Weights	Randomly chosen to be $\varepsilon_{\mathrm{Ex}}$ with probability p_{Ex}, $\varepsilon_{\mathrm{In}}$ otherwise
Delay	τ, fixed, homogeneous

D	Neuron and synapse model
Name	LIF Neuron
Type	Leaky integrate-and-fire neurons, δ-current input
Subthreshold dynamics	$\dot{V}_l(t) = -\gamma_l V_l(t) + \sum_f \left[\sigma\left(\sum_{j\in M_{\mathrm{Ex},l}(f)} \varepsilon_{lj}\right) + \sum_{j\in M_{\mathrm{In},l}(f)} \varepsilon_{lj}\right]\delta\left(t - t^f - \tau\right) + I_{0,l}$
Spiking	If $V_l(t^-) + \varepsilon \geq \Theta_{U,l}$ set $t^* = t$, emit spike with time stamp t^*, reset $V_l(t) = V_{\mathrm{r},l}$
Dendritic interactions	Modulation of simultaneous excitatory inputs with dendritic modulation function σ

E	Input
Type	Constant external input current
Description	Constant external suprathreshold input current $I_{0,l}$ to each neuron l

F	Measurements
Spiking activity as raster plots, spike rates, synchronous pulses and chains of synchronous pulses, distributions of membrane potentials	
